# Intrahost Diversity of Feline Coronavirus: A Consensus between the Circulating Virulent/Avirulent Strains and the Internal Mutation Hypotheses?

**DOI:** 10.1155/2013/572325

**Published:** 2013-03-27

**Authors:** Aline S. Hora, Karen M. Asano, Juliana M. Guerra, Ramon G. Mesquita, Paulo Maiorka, Leonardo J. Richtzenhain, Paulo E. Brandão

**Affiliations:** ^1^Department of Preventive Veterinary Medicine and Animal Health, College of Veterinary Medicine, University of São Paulo, Avenida Professor Dr. Orlando Marques de Paiva 87, Cidade Universitária, 05508-270 São Paulo, SP, Brazil; ^2^Coronavirus Research Group, College of Veterinary Medicine, University of São Paulo, Avenida Professor Dr. Orlando Marques de Paiva 87, Cidade Universitária, 05508-270 São Paulo, SP, Brazil; ^3^Department of Pathology, College of Veterinary Medicine, University of São Paulo, Avenida Professor Dr. Orlando Marques de Paiva 87, Cidade Universitária, 05508-270 São Paulo, SP, Brazil

## Abstract

To evaluate the most controversial issue concerning current feline coronavirus (FCoV) virology, the coexisting hypotheses of the intrahost and interhost origins of feline infectious peritonitis virus (FIPV) in regard to the pathogenesis of feline infectious peritonitis (FIP), this study aimed to assess the molecular diversity of the membrane gene FCoVs in 190 samples from 10 cats with signs of FIP and in 5 faecal samples from cats without signs of FIP. All samples from the non-FIP cats and 25.26% of the samples from the FIP cats were positive for the FCoV membrane (M) gene. Mutations in this gene consisted of SNP changes randomly scattered among the sequences; few mutations resulted in amino acid changes. No geographic pattern was observed. Of the cats without FIP that harboured FECoV, the amino acid sequence identities for the M gene were 100% among cats (Cats 1–3) from the same cattery, and the overall sequence identity for the M gene was ≥91%. In one cat, two different lineages of FCoV, one enteric and one systemic, were found that segregated apart in the M gene tree. In conclusion, the in vivo mutation transition hypothesis and the circulating high virulent-low virulent FCoV hypothesis have been found to be plausible according to the results obtained from sequencing the M gene.

## 1. Introduction

 Feline coronavirus (FCoV), a widespread pathogen of domestic cat populations worldwide, is an enveloped single-stranded RNA virus of the order Nidovirales, family Coronaviridae, subfamily Coronavirinae, genus *Alphacoronavirus, *species *alphacoronavirus 1*. Most infections are either asymptomatic or result in a mild, self-limiting gastrointestinal disease, and, in these cases, the causative agent is the feline enteric coronavirus (FECoV) pathotype. In contrast, feline infectious peritonitis (FIP), a possible complication of FCoV infection in a small proportion of cats, is a lethal, systemic immune-mediated disease caused by a second FCoV pathotype, feline infectious peritonitis virus (FIPV) [1, 2].

 FIP is characterised by fibrinous/granulomatous serositis, with protein-rich effusions in the body cavities of affected cats, as well as granulomatous-necrotising lesions, periphlebitis, and granulomatous inflammatory lesions in several organs, particularly in the liver, kidney, spleen, leptomeninges, and eyes [3]. The pathogenesis of FIP is not fully understood, but it has been shown that monocyte-triggered vasculitis, in association with systemic monocyte and endothelial cell activation, is an essential event [3], most likely in combination with antibody-mediated enhancement and complement activation [4]. Specific genetic determinants of these clinical outcomes have yet to be discovered in cats. This disease is one of the most serious viral infections in cats, not only because of its fatal nature but also because of the difficulties in diagnosing FIP antemortem and in controlling the spread of FCoV [5]. 

The key to a deeper understanding of FCoV diseases in cats is the exact nature of the relationship between the two FCoV pathotypes; two important hypotheses have been suggested. The hypothesis known as the “internal mutation theory” is widely accepted [1, 2, 6–9], and it states that FIP occurs when a cat is exposed to variants of FCoV that have mutated within the host and are able to disseminate from the gut (the primary site of infection) by gaining the ability to efficiently replicate within the macrophages [1, 2]. However, any stable genetic differences between FECoV and FIPV that can account for their differing pathogenicities remain to be identified [10]. 

The alternate “circulating high virulent-low virulent” FCoV hypothesis of viral pathogenesis suggests that both distinctive pathogenic and benign lineages of FECoV might be present in a cat population and that the disease will develop only in those cats infected by the virulent strains already available from other infected cats [5]. The existence of distinct “high virulent-low virulent FCoVs” is an alternate and less popular hypothesis for FIP pathogenesis [5, 10]. As FIP occurs sporadically and outbreaks of FIP in domestic cat populations are rather uncommon, there has been little epidemiologic support for this hypothesis [1]. 

Most authors have concurred that although a low-level monocyte-associated viraemia is found with FECoV infections, this virus is mainly confined to the gut [10, 11]. This is in contrast to the highly virulent FIPV, which disseminates systemically with high viral titres. Thus, obtaining sequence data from enteric and nonenteric FCoVs found within individual cats with FIP may shed more light on any genetic differences between FECoV and FIPV [10]. 

To evaluate the most controversial issue concerning current FCoV virology, the coexisting hypotheses of the intrahost and interhost origins of FIPV in regard to FIP pathogenesis and to gain more insight into FCoV evolution, this study aimed to assess the molecular diversity of FCoVs in multiple organs of cats with signs of FIP and in faecal samples from cats without signs of FIP.

## 2. Materials and Methods

### 2.1. Animals and Sample Collection

During 2010–2012, tissue samples (eye, cerebrum, cerebellum, lung, heart muscle, thoracic lymph node, thymus, liver, spleen, stomach, mesenteric lymph node, peripancreatic lymph node, kidneys, large and small intestines, and urinary bladder), abdominal, thoracic, and pericardial effusions, aqueous humour, and faecal samples were collected at necropsies from 10 deceased or euthanised unrelated cats with suspected FIP, for a total of 190 samples. Sterile instruments and disposable materials were used for sample collection.

Additionally, faecal samples from five cats without clinical signs of FIP were also collected; none of these had cats developed FIP as of the time of writing this study. These cats came from 3 multiple cat households, one of them with an outbreak of diarrhoea at the time that the 3 samples from the different cats were collected. All samples were flash-frozen in liquid nitrogen and stored at –80 °C until the RNA extraction.

### 2.2. Total RNA Extraction

Organ samples were prepared as 30% (v/v) suspensions in UltraPure diethyl pyrocarbonate-(DEPC-) treated water (Invitrogen, Carlsbad, CA, USA), submitted to 3 freeze-thaw cycles in liquid nitrogen at 56°C, and clarified at 5,000 ×g for 15 min at 4°C. Effusion and aqueous humour were concentrated at 12,000 ×g for 15 min at 4°C. Faecal samples were prepared as 30% (v/v) suspensions in UltraPure diethyl pyrocarbonate-(DEPC-) treated water (Invitrogen, Carlsbad, CA, USA), clarified at 12,000 ×g for 15 min at 4°C. The total RNA was extracted from the supernatants of organs and faecal suspensions and pellets from effusions with TRIzol reagent (Invitrogen, Carlsbad, CA, USA), according to the manufacturer's instructions. The RNA was eluted in 30 *μ*L of UltraPure DEPC-treated water.

### 2.3. RT-PCR for the Detection of the FCoV mRNA of the Membrane (M) Gene

All samples were screened for the presence of FCoV M gene mRNA using primers previously described [12], with modifications (2RNAmA TAATRMCATARACGADCCAGCT, nt 26440–26461, and 2RNAmS GTGCTAGVTTTGTCTTCGGACAMC, nt 60–83, positions regarding strain FIPV 79–1179). 

 A positive RNA control was prepared from the abdominal effusion of a cat with FIP by an in vitro transcription with a MEGAscript T7 kit (Ambion, Austin, TX, USA) according to the manufacturers' instructions, using the above-mentioned primers. UltraPure DEPC-treated water (Invitrogen, Carlsbad, CA, USA) was used as a negative control.

For the 190 samples, the cDNA was synthesised in 10 *μ*L reactions using 3.5 *μ*L of the RNA solution and 1 *μ*M 2RNAmS of the reverse primer and M-MLV Reverse Transcriptase (Invitrogen, Carlsbad, CA, USA), as per the manufacturer's instructions. 

The subgenomic mRNA of the M gene was then amplified using 0.5 *μ*M of each primer and GoTaq Green Master Mix 1X (Promega Corporation, Madison, WI, USA) according to the manufacturer's protocols. The primer annealing temperature was 50°C. The PCR products (295 bp) were visualised by electrophoresis on 1.5% agarose gels stained with SYBR Green I nucleic acid gel stain (Invitrogen, Carlsbad, CA, USA).

### 2.4. Partial M Gene Amplification and Sequencing

cDNA was synthesised using Random Primers (Invitrogen, Carlsbad, CA, USA) and M-MLV Reverse Transcriptase (Invitrogen, Carlsbad, CA, USA), according to the manufacturer's protocols. The partial M gene (575 bp) was then amplified using 0.5 *μ*M of both reverse (26925–26944 nt positions regarding strain FCoV UU47) and forward primers (26968–26292 nt positions regarding strain FCoV UU47) [5] and GoTaq Green Master Mix 1X (Promega Corporation, Madison, WI, USA) as per the manufacturer's instructions. 

Amplicons were purified from agarose gels with Illustra (GE Healthcare, Buckinghamshire, UK) and submitted to bidirectional DNA sequencing with BigDye 3.1 (Applied Biosystems, Carlsbad, CA, USA), according to the manufacturer's protocols. Products were resolved using a 3500 Genetic Analyzer (Applied Biosystems, Foster City, CA, USA), and the chromatograms were analysed with Phred at http://asparagin.cenargen.embrapa.br/phph/. Positions with a quality score >20 were used to generate contiguous sequences with Cap-contig implemented in the software BioEdit 7.0.9.0 [13]. Those sequences were then submitted to BLAST/n at http://www.ncbi.nlm.nih.gov/BLAST to confirm the Amplicon identities.

**Figure 1:**
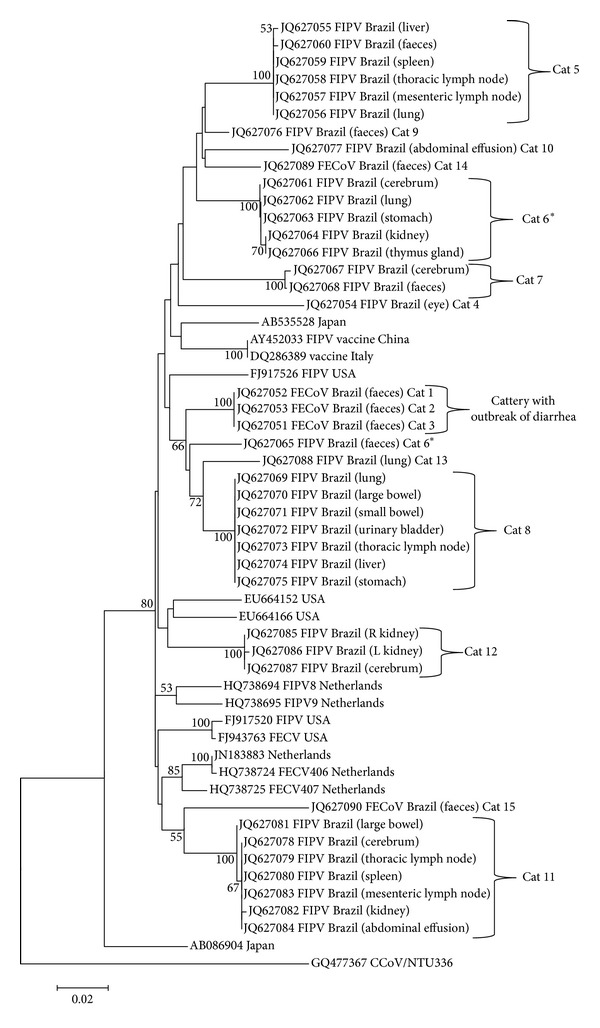
Neighbour-joining MCL phylogenetic tree of the M gene partial sequences of FCoV (positions from 26293 to 26907 regarding strain FCoV UU47). The tree was constructed using a canine coronavirus sequence as an outgroup (GQ477367). Numbers on the nodes indicate the bootstrap support from 1,000 replications. Only bootstrap values >50 are shown. The scale bar represents the number of substitutions per nucleotide. The numbers from 1 to 15 identify each cat.

The M gene partial sequences and the putative amino acid sequences from each sample were aligned with homologous sequences from both FCoVs pathotypes retrieved from GenBank (Figure 1) with CLUSTAL/W in BioEdit 7.0.9.0 [13], and a phylogenetic tree for the nucleotide sequences was generated with the neighbour-joining distance algorithm and the maximum composite likelihood model with 1,000 bootstrap replicates using MEGA 5.0 [14]. Amino acid alignment was used to search for previously reported pathotype-specific markers [5].

### 2.5. Histopathologic Analysis

Organ samples were also fixed in 10% buffered formalin and routinely embedded in paraffin. Sections (5 *μ*m) were stained with hematoxylin and eosin (HE). The HE-stained slides of the organ sections were evaluated for evidence of granulomatous and pyogranulomatous lesions.

## 3. Results

### 3.1. RT-PCR for the Detection of FCoV mRNA of the Membrane Gene

In all 10 cats with FIP, FCoV mRNA was detected in at least one of the samples. Of the 190 individual samples, 77 (40.53%) were positive for the M gene. The abdominal effusions (5/6), mesenteric lymph nodes (7/10), large intestines (7/10), lungs (6/10), thoracic effusions (4/8), kidneys (10/20), and aqueous humours (8/20) were the most frequent FCoV replication sites. Of the 5 faecal samples from cats without FIP, 3 were positive for FCoV M gene mRNA. 

### 3.2. Partial M Gene Amplification, Sequencing, and Phylogenic Analysis

Of the euthanised or diseased cats, 25.26% samples (48/190) were positive for the FCoV M gene. Multiple samples from each cat were determined to be positive, by RT-PCR, for the FCoV M gene. Of these, the most frequently positive samples were the contents of the large intestine (5/11), spleen (4/10), and lungs (4/10). All faecal samples from the 5 cats without FIP were positive for the FCoV M gene.

The sequences obtained in this study were submitted to the GenBank database under the accession numbers JQ627051-JQ627090.

When compared by phylogenic analysis, the nucleotide sequences of the M gene (positions from 26293 to 26907 regarding strain FCoV UU47) from the cats with FIP and the cats with FECoV in this study were distributed in paraphyletic groups (Figure 1). Sequence strains from 3 cats with diarrhoea but without PIF (Cats 1–3) from a multiple cat household were grouped in the same cluster. The sample-specific FCoV strain's differentiation and nucleotide polymorphism among sequences from the same cat were low (≥98%), whereas the overall sequence identification for the M gene was ≥91% among all FIPV sequences (Table 1). Mutations consisted of minor SNP changes that appeared to be randomly scattered among the sequences; few mutations resulted in amino acid changes. No geographic pattern was observed. Of the cats without FIP that harboured FECoV, the amino acid sequence identities for the M gene were 100% among cats (Cats 1–3) from the same cattery, and the overall sequence identity for the M gene was ≥91%. 

Nonetheless, a striking exception was Cat 6, in which two different lineages of FCoV, one enteric and one systemic, were found that segregated apart in the M gene tree (Figure 1), with a nucleotide identity of 94% among the enteric sequence and the systemic (*n* = 5) sequences.

**Figure 2:**
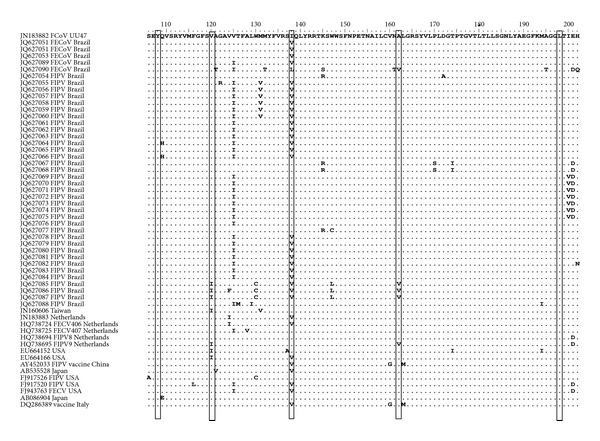
Alignment of the amino acid sequences of partial M proteins of the FCoVs compared with the feline coronavirus reference sequence (GenBank accession number JN183882) and other FCoV sequences from GenBank. The 5 aa residues at positions 108, 120, 138, 162, and 198 suggested as potential diagnostic sites [5] are boxed.

Five amino acid sites in the M protein suggested as potential FIPV signatures [5] were evaluated based on the reference sequence for FCoV (GenBank JN183882). Within the lineages obtained in the present study, no amino acid polymorphism was observed at position 108 or 198 (Figure 2). The amino acids at positions 120, 138, and 162 occurred with no specific pattern in the FIPV and FECoV sequences: Val120 in 100% (5/5) of FECoV and in 91.4% (32/35) of FIPV; Ile120 in 8.6% (3/35) of FIPV; Ile138 in 34.3% (12/35) of FIPV; Val138 in 80% (4/5) of FECoV and 65.7% (23/35) of FIPV; Leu138 in 20% (1/5) of FECoV; Ala162 80% (4/5) of FECoV and in 91.4% (32/35) of FIPV; Val162 in 20% (1/5) of FECoV and 8.6% (3/35) of FIPV.

### 3.3. Histopathologic Analysis

A histopathological analysis was performed for 8 of the 10 necropsied cats. HE-stained sections typically showed localised inflammation with macrophages, neutrophils, lymphocytes, and plasma cells. Vascular lesions were found surrounded by a proliferation of inflammatory cells. Focal accumulations of inflammatory cells and necrotic-proliferative lesions were observed in granulomatous lesions.  Table 2 shows the main histopathological features and the FCoV replication sites for each cat.

## 4. Discussion and Conclusions

In this investigation, both double and single FCoV pathotype infections have been found in cats with feline infectious peritonitis, which supports both hypotheses of a viral pathogenesis of the disease based on the M gene phylogeny from FCoV strains detected in naturally infected cats.

A phylogenetic analysis showed that nucleotide sequences of the FIPV and FECoV M genes do not segregate in a biotypical pattern, a distribution consistent with the in vivo mutation transition hypothesis, which postulates that a de novo virus mutation occurs in vivo, giving rise to highly virulent strains without the need for exogenous highly virulent strains. A similar pattern was observed in a previous study that also examined the M gene [8] which determined that FIPVs originate from FECVs by the accumulation and selection of point mutations [1, 2, 7]. The internal mutation hypothesis has been widely accepted and is mainly supported by the close similarities between FECoV and FIPV and the low incidence of FIP outbreaks, despite the high proportion of FCoV-seropositive cats [6, 15–17]. 

Although no disease-specific clusters of FCoVs were found, samples from Cat 6 were particularly informative, as two different strains of FCoV, one enteric and one systemic, can be clearly observed in Figure 1, which, in contrast to the other sampled individuals, is in agreement with the two different pathotypes hypothesis. The source for different FCoVs lineages in a cat may be a superinfection [5], but in an experimental study in which cats were infected with two variants of FIPV, a variant prevailed in each cat [7]. Cat 6 might thus have acquired at least two lineages, one with tropism for the intestinal epithelium and the other with a macrophage tropism and a systemic spread that led to the development of the disease.

The full length genomic sequence of the viruses found in two different tissues of a cat with classical FIP, one enteric (jejunum) and one nonenteric (liver), revealed a 100% nucleotide identity [10], a finding that questions the well accepted “internal mutation theory” of FIPV pathogenicity. Nonetheless, it must be taken into account that consensus sequencing, without prior cloning, can mask minority virus populations, and further viral lineages could be present at lower levels within an animal. The M gene mRNA that was detected in the faeces from Cat 6 is a further indication that this cat had two active infections, one systemic and the other enteric.

The current belief is that cats with FIPV do not transmit this pathotype to other cats [2], but it is theoretically possible for cats with an infection in the intestinal wall or kidneys to shed FIPV in the faeces or urine, respectively. The identity among the sequences obtained from the stool or intestines with the sequences of other tissues in cats with FIP in this study was ≥99.9%, indicating that the FIPV pathotype can indeed be spread via faeces and that a cat with FIP can excrete FCoV strains with no distinction between FECoV and FIPV. Accordingly, mutations in the 3c gene identical to the FIPVs from tissues were present in the faeces of some cats, thus making horizontal transmission theoretically possible in certain circumstances [7, 18] and supporting the two-pathotype hypothesis. 

A sample of the urinary bladder from a cat (Cat 8) was positive for M gene mRNA; this finding shows that the urinary shedding of FCoV is also plausible. In cats, the infectivity of urine for FCoV has previously been reported [19]. Likewise, human patients with severe acute respiratory syndrome (SARS) caused by the HCoV-SARS coronavirus have been reported to shed the virus via urine and faeces [20, 21]. Furthermore, avian coronavirus also displays faecal excretion and replication in the kidneys of domestic fowl [22]. These reports not only show that FCoV might be shed via the urinary tract but also that this is a conserved feature for coronaviruses. 

The phylogenetic analysis has shown that the nucleotide sequences of FCoV generally clustered according to the cattery, irrespective of their pathotypes [9]. In some circumstances, multiple FCoVs lineages can be observed in the same cattery, due to the elevated admission of new individuals in cases of open catteries or shelters [8]. A unique genetic fingerprint was observed in sequences obtained from cats (Cats 1–3) of the same cattery that underwent an outbreak of diarrhoea, indicating that coronavirus infection most likely originated from a single founder virus within this closed group of cats. Catteries and closed multicat environments usually have one major enzootic strain of coronavirus that persists over long periods of time, and these major enzootic strains are dominant even with exposure to other strains [1, 2, 6, 7, 23]. 

A phylogeographic pattern was not observed among the FCoVs studied herein compared with sequences from other countries, but a pronounced mutational drift (7–10%) was found in strains from the same geographic region, quite similar to the 6–16% reported for cats from distant areas of the western USA [7]. As this is the first report on FCoV diversity in Brazilian cats, further comparisons with other geographic areas within the country are an issue for future research. Recombination and a high mutation rate are common phenomena among coronaviruses, which provide a mechanism for the rapid emergence of new viral strains with dramatically altered tropisms and pathogenicity, which can have a significant impact on the host disease [2, 5–7, 9].

FCoV strains from FIP-positive cats, as determined by a classical pathological or immunohistochemistry diagnosis, have been reported as displaying YIVAL/YIIAL in the M protein (positions 108, 120, 138, 162, and 198) [5], but such markers were absent in all FECoV and FIPV putative M sequences recovered in the present investigation, as previously described [8, 18], which strongly argues against the currently accepted criteria for FIPV/FECoV differentiation.

As both hypotheses have been found as plausible according to these results, this could mean that FIPV can both emerge endogenously and be transmitted to different cats as a “ready” virulent pathotype, which has major implications for the understanding of the dynamics of viral transmission. 

## Figures and Tables

**Table 1:** Feline coronavirus membrane (M) gene sequence identities (positions from 26293 to 26907 regarding strain FCoV UU47) from different samples of a same cat with FIP.

Cat	% sequence identity^a^	SD	Number of sequences	GenBank accession number
Cat 5	99.9	0.001	*n* = 6	JQ627055-60
Cat 6	98.0	0.028	*n* = 6	JQ627061-66
Cat 7	99.6	0.000	*n* = 2	JQ627067-68
Cat 8	100.0	0.000	*n* = 7	JQ627069-75
Cat 11	99.9	0.001	*n* = 7	JQ627078-84
Cat 12	99.9	0.001	*n* = 3	JQ627085-87

^a^The percentage of the sequence identity was determined by comparison with the consensus sequences of the FCoV obtained from different samples of the same cat. SD: standard deviation.

**Table 2:** Histopathological analysis and results of FCoV mRNA RT-PCR in diseased cats with FIP.

Cat (*n* = 8)	Histopathological findings	Positive samples for mRNA FCoV
Cat 6	Pyogranulomas in lung, kidneys, cerebrum, and cerebellum Mild-to-moderate, subacute, multifocal fibrinous pleuritis Vasculitis in heart, lung, omentum, kidneys, cerebrum, and cerebellum	Cerebrum, cerebellum, lung, thymus, spleen, stomach, mesenteric lymph node, kidneys, aqueous humour, and faeces

Cat 7	Pyogranulomas in liver, kidneys, cerebrum, and cerebellum Vasculitis in cerebrum	Cerebellum, large intestine, and large intestine content

Cat 8	Severe, subacute, diffuse fibrinous pleuritis and peritonitis	Lung, thoracic lymph node, thymus, liver, spleen, stomach, mesenteric lymph node, peripancreatic lymph node, kidneys, large and small intestines, small intestine content, urinary bladder, and abdominal effusion

Cat 9	Diffuse, severe, nonsuppurative meningoencephalitis	Aqueous humour right eye, large intestine, and large intestine content

Cat 10	Moderate, subacute, diffuse fibrinous pleuritis	Mesenteric lymph node, large and small intestines contents, and abdominal effusion

Cat 11	Subacute, multifocal fibrinous peritonitis Moderate, diffuse interstitial pneumonia Pyogranulomas in kidneys (with bacteria) and liver	Cerebrum, aqueous humour left eye, mesenteric lymph node, and large intestine

Cat 12	Subacute, focally extensive fibrinous pleuritis Pyogranulomas in kidneys	Cerebrum, cerebellum, aqueous humour left eye, and thoracic effusion

Cat 13	Moderate, multifocal granulomatous pleuritis Severe, granulomatous, interstitial nephritis, associated with coalescing areas of necrosis	Cerebrum, aqueous humour left eye, lung, thoracic effusion, mesenteric lymph node, large and small intestines contents, kidneys, and abdominal effusion
